# Editorial: Emerging trends in large-scale data analysis for neuroscience research

**DOI:** 10.3389/fninf.2024.1538787

**Published:** 2024-12-20

**Authors:** Farouk S. Nathoo, Olave E. Krigolson, Fang Wang

**Affiliations:** ^1^Department of Mathematics and Statistics, University of Victoria, Victoria, BC, Canada; ^2^Exercise Science, Physical and Health Education, University of Victoria, Victoria, BC, Canada; ^3^Department of Computer Science, Brunel University London, London, United Kingdom

**Keywords:** neuroimaging data analysis, fMRI, computational modeling, big data, machine learning

Neuroscience has witnessed a surge in data generation due to advancements in experimental techniques like electrophysiology, imaging, and genomics. To gain deeper insights into the brain's structure and function in health and disease, it has become essential to conduct large-scale data analyses.

Analyzing large datasets in neuroscience offers various applications, such as uncovering patterns in neuronal activity, building theoretical models, and predicting behavior. This has created an increasing demand for scalable, efficient, and robust data analysis and machine-learning methods that can handle the vast volume of data generated. By collaborating with domain experts, this research initiative seeks to push the frontiers of large-scale data analysis in neuroscience and foster innovative discussions to meet the field's emerging needs.

The primary aim of this Research Topic is to showcase recent progress in data-driven approaches for studying the brain. It focuses on tackling challenges in managing, processing, and interpreting large-scale neuroscience data while identifying future research opportunities. This Research Topic will delve into state-of-the-art tools and methods for analyzing, integrating, and interpreting extensive neuroscience datasets.

Excluding the retraction, there are five papers published in this Research Topic. Hsu et al. consider the problem of warping and registering brain images to a standard template, which can introduce spatial errors and reduce accuracy. They develop LYNSU (Locating by YOLO and Segmenting by U-Net), an automated method for segmenting neuropils in fluorescence images from the FlyCircuit database, eliminating the need for warping and facilitating high-throughput anatomical analysis and connectomics in the Drosophila brain. They demonstrate performance comparable to manual annotations, with a 3D Intersection-over-Union (IoU) of 0.869, and segments a neuropil in about 7 seconds.

Miranda considers task-based fMRI studies and develops a fast Bayesian function-on-scalar model to estimate population-level activation maps for the working memory task. The proposed approach uses a canonical polyadic (CP) tensor decomposition to extract shared and subject-specific features from individual coefficient maps. The subject-specific features are modeled as functions of covariates within a Bayesian framework that accounts for correlations in the CP-extracted features. The proposed decomposition facilitates fast computation and allows efficient MCMC estimation of population-level activation maps.

Dang et al. consider the problem of decoding and feature selection in high dimensions. They introduce the optimized Forward Variable Selection Decoder (oFVSD) toolbox as a feature selection methodology that combines forward variable selection (FVS) and hyperparameter optimization integrated with 18 machine learning models. They test sex classification and age range regression on 1,113 structural MRI datasets and demonstrate performance improvements over models without FVS. The methodology is available as an open-source Python package.

Bologna et al. consider the construction of data-driven brain models using neural simulation environments and large-scale computing facilities. They developed the EBRAINS Hodgkin-Huxley Neuron Builder (HHNB), a web resource for building single cell neural models via the extraction of activity features from electrophysiological data with estimation based on a genetic algorithm. HHNB then allows simulation of the brain model using the estimated model through an interactive setting.

Kim et al. consider the visualization of gene expression obtained using RNA sequencing across the brain. Molecular patterns emerging from spatial transcriptomic data can be associated with circuitry and function in the neocortex. They propose a web app LaminaRGeneVis for visualizing laminar gene expression across datasets collected using bulk, single-nucleus, and spatial RNA sequencing. Allowing for normalizations across different datasets, the app supports single- and multi-gene analyses, data visualization and statistics for the adult human neocortex.

